# Deep Ego-Motion Classifiers for Compound Eye Cameras

**DOI:** 10.3390/s19235275

**Published:** 2019-11-29

**Authors:** Hwiyeon Yoo, Geonho Cha, Songhwai Oh

**Affiliations:** Department of Eletrical and Computer Engineering and ASRI, Seoul National University, 1 Gwanak-ro, Gwanak-gu, Seoul 08826, Korea; hwiyeon.yoo@rllab.snu.ac.kr (H.Y.); geonho.cha@rllab.snu.ac.kr (G.C.)

**Keywords:** Bio-inspired structure, compound eye camera, compound image, ego-motion classification

## Abstract

Compound eyes, also known as insect eyes, have a unique structure. They have a hemispheric surface, and a lot of single eyes are deployed regularly on the surface. Thanks to this unique form, using the compound images has several advantages, such as a large field of view (FOV) with low aberrations. We can exploit these benefits in high-level vision applications, such as object recognition, or semantic segmentation for a moving robot, by emulating the compound images that describe the captured scenes from compound eye cameras. In this paper, to the best of our knowledge, we propose the first convolutional neural network (CNN)-based ego-motion classification algorithm designed for the compound eye structure. To achieve this, we introduce a voting-based approach that fully utilizes one of the unique features of compound images, specifically, the compound images consist of a lot of single eye images. The proposed method classifies a number of local motions by CNN, and these local classifications which represent the motions of each single eye image, are aggregated to the final classification by a voting procedure. For the experiments, we collected a new dataset for compound eye camera ego-motion classification which contains scenes of the inside and outside of a certain building. The samples of the proposed dataset consist of two consequent emulated compound images and the corresponding ego-motion class. The experimental results show that the proposed method has achieved the classification accuracy of 85.0%, which is superior compared to the baselines on the proposed dataset. Also, the proposed model is light-weight compared to the conventional CNN-based image recognition algorithms such as AlexNet, ResNet50, and MobileNetV2.

## 1. Introduction

A compound eye, which is commonly known as an insect eye, has a remarkably sophisticated structure. It has a hemispherical surface and a large number of single eyes are deployed regularly on the hemispherical surface. Here, each single eye observes a low resolution scene in different angles of small field of views (FOV). The compound eye is a union of the single eyes so that it can observe high-resolution scenes with a large FOV. Thanks to its unique structure, the compound eye has various benefits such as a large FOV, low aberrations, and a large depth of field [1,2,3,4].

Having been inspired by these interesting characters of the compound eye structure, many researchers have tried to develop artificial compound eye cameras [4,5,6,7]. Most of these researches mainly aimed to develop a micro-sized camera hardware which mimics the structure and function of the real compound eyes of insects. Meanwhile, there also have been researches that focused on developing computer vision algorithms for compound images, such as high-resolution image reconstruction [8], objectness estimation [9], semantic segmentation [10], and ego-motion estimation [11]. Specifically, [9,10] emulate compound images from RGB image sources rather than directly capture scenes using the hardware such as [4,5,6,7]. The emulated compound image consists of a large number of small single eye images, similar to the case of real compound eyes. A single eye image in the emulation is a low resolution RGB image with a small FOV, and a set of different angled single eye images constitute a high resolution compound image of a large FOV. Since a large-scale dataset can be collected easily by the emulation, these emulated compound images can be utilized in CNN-based high-level computer vision applications, such as objectness estimation [9] and semantic segmentation [10].

In this paper, to the best of our knowledge, we tackle the problem of ego-motion classification for compound eye cameras using convolutional neural network (CNN) for the first time, of which the goal is to classify the motion of compound eye camera given two consecutive emulated high resolution compound images. The ego-motion classification is the problem that focuses on the movement of the camera itself, which is different from the previous camera motion estimating algorithms that aim to estimate the transition between two scenes [12,13,14,15,16,17]. Here, the ego-motion classification of the compound eye camera gives an important contribution to the robot community, since knowing the moving direction of a robot is critical for problems such as localization and navigation.

For the input of the proposed algorithm, we first emulate the high resolution compound images based on the compound image mapping method proposed in [10]. Different from the previous emulated compound-image-based works [9,10], we focus on the characteristic that the compound image consists of a lot of single eye images. The two sequentially consecutive compound images are fed into the proposed CNN-based ego-motion classification network, and the network outputs local motion classes for each single eye image. The final ego-motion classification is obtained by voting of these local classifications. A thing to note here is that the advantage from the voting-based strategy enables us to design the local classification algorithm focusing more on the computational efficiency rather than the accuracy.

To evaluate the proposed method, we propose a new ego-motion classification dataset for compound eye cameras, which are based on videos collected in the inside and outside of a certain building. The proposed dataset consists of consecutive compound images which capture scenes such as classroom, aisle, stairs, and terrace, and the corresponding ground truth ego-motion. More details of the proposed dataset are described in Section 4.2. In the experiments, we have tested the proposed framework with various pixel sizes of single eye images and receptive field of local classification, to find an appropriate configuration of compound eye camera for the ego-motion classification.

The contributions of the paper are summarized as:

- We propose a compound eye ego-motion classification algorithm using CNN for the first time.
- We introduce a new dataset for the compound eye ego-motion classification.
- We analyze the effect of the size of a single eye image and receptive field size of local classification to the performance of the algorithm.

The remainder of this paper is organized as follows. In Section 2, related work is introduced. The proposed low complexity compound eye ego-motion classification network is introduced in Section 3. The proposed dataset and baseline methods are described in Section 4. The experimental results are described in Section 5, and we conclude the paper in Section 6.

## 2. Related Work

### 2.1. Compound-Image-Based Application

There have been some compound-image-based applications that utilize the unique structure of compound images [8,9,10,11]. A high-resolution image reconstruction method from low-resolution single-eye-images was developed in [8]. They rearranged pixels in all single-eye images in a virtual image plane consisting of fine pixels. Neumann et al. [11] proposed a compound-image-based 3D ego-motion estimation method. They showed that the geometry of the compound eye is optimal for 3D ego-motion estimation, and a linear camera motion estimation algorithm was proposed. However, these works focused on low-level applications such as super-resolution [18] and depth estimation [19], that are difficult to use for general recognition problems. Some high-level vision applications which use deep neural network, have been proposed in [9,10]. Yoo et al. [9] proposed an objectness estimation framework based on compound images. Cha et al. [10] proposed a semantic segmentation method for compound images.

### 2.2. Camera Motion Estimation

There have been several studies for estimating camera motion between two images [12,13,14,15]. Especially, block-based matching algorithms are standard techniques for motion estimation in video sequences [12]. Such block-based matching methods divide an image frame into non-overlapping blocks, and search for the best matching block in the next frame for each block. The motion vector is defined as the relative displacement between a reference block and the matching block in the comparing frame. The camera motion is estimated based on these motion vectors of the blocks.

Moreover to the RGB or RGB-D image space, there also have been some researches about motion estimation for unique image space such as fisheye video sequence [16], and omnidirectional image space [17]. Eichenseer et al. [16] proposed a hybrid block-based motion estimation method for real-world fisheye videos by combining perspective projection with considerations about the fisheye image structure. Simone et al. [17] presented an extension of block-based motion estimation for panoramic omnidirectional image sequences by considering spherical geometry of the imaging system. These researches focus on unique domains such as fisheye video and panoramic omnidirectional image sequences, so they are not applicable to the compound image domain which we are target for.

In recent days, deep-neural-network-based camera motion estimation algorithms have been studied [20,21,22]. Costante et al. [20] adopted convolutional neural network (CNN) to solve visual odometry. They used CNN to extract visual features for achieving frame to frame ego-motion estimation. Ummenhofer et al. [21] proposed algorithm called DeMoN, which estimated depth and camera motion from two successive RGB images. The DeMoN architecture used multiple stacked CNN-based-encoder-decoder networks for the estimation. Du et al. [22] proposed an end-to-end deep model to solve ego-motion classification of the driving vehicle with video sequences. They used CNNs for extracting visual features from the video frames and used Long Short Term Memory (LSTM) to model the temporal correlation of the video sequences. Also, there have been several studies that combine the CNN-based learning method and the block-based, or patch-based attention strategy to solve computer vision problems other than the camera motion estimation, such as abnormal event detection [23], disease recognition [24], video restoration [25], and facial expression recognition [26].

In spite of their great performances due to the power of deep neural networks, these previous works were studied only in RGB, or RGB-D image space. The compound image, which is the unique image domain that we tackle in this paper, has a spherical surface for visual perception. Therefore, plane RGB or RGB-D image based algorithms are not appropirate to deal with our problem.

Moreover, these previous works for estimating camera motion aim to estimate the transition between two scenes. On the other hand, the ego-motion classification that we aim to, focuses on the movement of the camera itself, so the previous works is not directly compatible to the ego-motion classification.

## 3. Compound Eye Ego-Motion Classification Network

In this section, we introduce the proposed compound eye ego-motion classification network. We use the compound image mapping method proposed in [10] to construct compound image $I\in R^{3S^{2}\times n_{s}}$ from RGB image, where *S* and $n_{s}$ are the size and the total number of single eye images, respectively. An unfortunate fact is that the raw compound images are hard to be applied to the powerful conventional CNNs designed for RGB images due to its unique structures. To resolve this issue, we preprocess the raw compound images based on the method explained in Section 3.1. The preprocessed compound images are utilized in the proposed network which is introduced in Section 3.2 and Section 3.3.

### 3.1. Vectorized Compound Images

**Figure 1:**
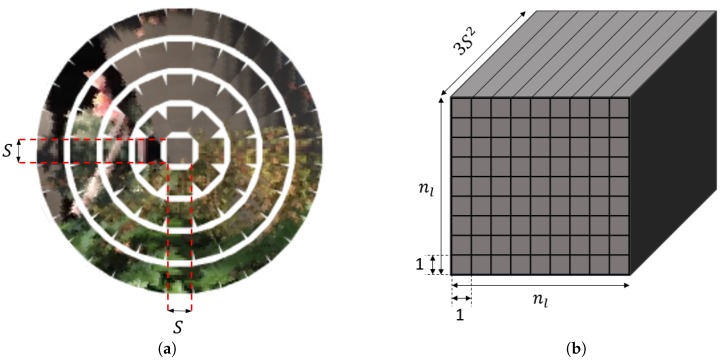
An illustration of the compound image vectorization. Constrained compound images can be transformed to tensor representations of multi-dimensional 2D images. (**a**) Each single eye image has the size of S×S, and reshaped into a vector of dimension $R^{3S^{2}}$. (**b**) The whole compound image is reshaped into a tensor of dimension $R^{n_{l}\times n_{l}\times 3S^{2}}$. Here, $n_{l}$ is the size of the transformed tensor.

Conventional CNNs are not suitable for processing compound images since they assume spatially-continuous inputs, which is not the case for compound images. To alleviate this issue, we transform the input compound images to tensor representations of multi-dimensional 2D images, so that we can utilize CNN structure by changing the channel size of input filter. For the transformation, we put few constraints to the compound image configuration. We assume that there are discrete levels on the hemisphere surface and each level has uniformly increasing polar angle. The number of single eye images at the *l*th level is (8l−8) except the first level, which has only one single eye image at the center. In this configuration, we can transform compound images in a square-form tensor of which each pixel represents a vectorized single eye image. As a result, the compound image data can be transformed to a tensor representation of $R^{n_{l}\times n_{l}\times 3S^{2}}$, where $n_{l}$ is the size of the transformed tensor. We call this tensor representation of compoun image as a vectorized compound image. An example of the vectorization process is visualized in Figure 1. Note that the vectorized compound image not only facilitates the use of the power of CNN, but also preserves the neighboring structures of the original compound images. In particular, neighboring-structure-preserving property is crucial for compound-image-based applications as it is one of the unique features of compound images.

### 3.2. Local Motion Classification

The proposed framework utilizes one of the unique features of compound images that they consist of numerous single eye images. Specifically, we aggregate local motion classes, which are obtained from single eye images, for the final ego-motion classification. In this section, we introduce the proposed local motion classification scheme.

**Figure 2:**
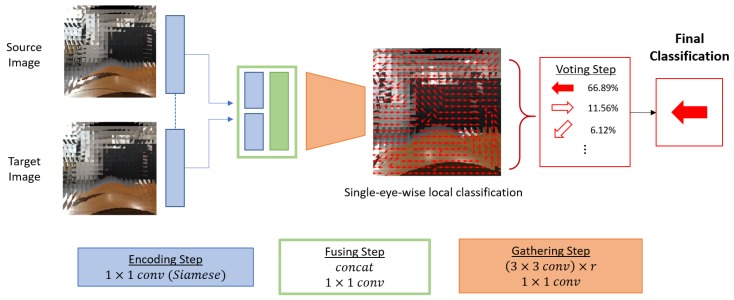
Overview of the proposed compound eye ego-motion classification network. The whole structure consists of four steps: the *encoding step*, the *fusing step*, the *gathering step*, and the *voting step*. The *encoding step* consists of a 1×1 convolutional layer with Siamese network structure to encode two inputs in the same manner. In the *fusing step*, encoded features from two inputs are concatenated and fused by a 1×1 convolutional layer. In the *gathering step*, 3×3 convolutional layers are applied to widen the receptive field of a local classification. *r* is the number of stacked 3×3 convolutional layers which determines the range of the receptive field of varying configurations. At the end of the *gathering step*, local motion class of each single eye is obtained by a 1×1 convolution. The *voting step* determines the final classification by finding the mode class among the local motion classifications.

**Figure 3:**
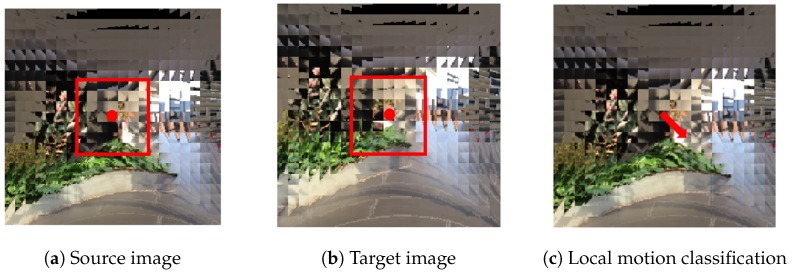
(**a**,**b**): A visualization of the receptive field of a single-eye-wise local motion classification with r=3. (**c**): A single-eye-wise local motion classification from compound images.

Inputs of the local motion classifier are two temporally-consecutive vectorized compound images. The two inputs are propagated through the proposed network which consists of three steps: the *encoding step*, the *fusing step*, and the *gathering step* (see Figure 2). First, in the *encoding step*, we incorporate a 1×1 convolution to both of the two inputs. Here, we adopt the Siamese network structure [27], which encodes two inputs in the same manner for estimating whether two inputs represent the same object or not. We use this encoding strategy of the Siamese network to encode each single eye image in the same manner. Then in the *fusing step*, the encoded features from the two compound image inputs are concatenated, and encoded again with a 1×1 convolution to fuse information of the two inputs. After that, *r* number of 3×3 convolutional layers are stacked at the *gathering step* to gather spatial information of the neighboring single eye images. With this neighbor information, the last layer of the *gathering step* outputs local motion class for each single eye image with a 1×1 convolution. Here, the receptive field size of the local classification becomes (2r+1)×(2r+1), since all 3×3 convolutional layers in the *gathering step* have stride of 1 and zero padding of 1. For example, the receptive field of the local classification is 7×7 if r=3. Figure 3a,b show an example of the receptive field of a single eye image on an input pair. The detailed architecture of the proposed network is described in Table 1.

**Figure 4:**
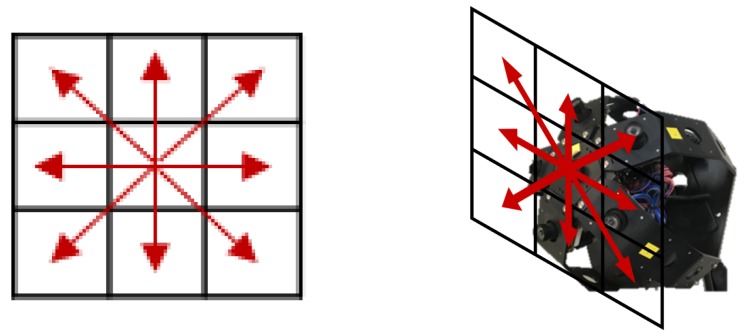
Discretized camera motion in eight directions. The discretized camera motion space is a subset of 2D plane which is tangent to the center of the camera. The right image illustrates the discretized camera motion with the compound eye camera.

To reduce the computational complexity of the proposed framework, we discretize the camera movement space in $n_{d}$ classes. We split two dimensional camera motion in eight directions; up, down, left, right, up-left, up-right, down-left, and down-right, which makes $n_{d}=8$. The discretized directions are illustrated in Figure 4. Finally, the probability distribution of each direction is obtained by a softmax operation over the output of the *gathering step*. Figure 3c shows an example of local motion classification of a single eye image position. Detailed structure of the proposed network is represented in Table 1.

### 3.3. Aggregation of Local Classifications

So far, we have obtained local motion classes based on each of the single eye position. Since single eyes are rigidly located on the compound eye camera hemisphere surface, all single eyes should have the identical motion when the camera moves. However, a single eye motion classification only relies on its neighboring single eye features, so the result can be different from the true movement of the entire compound eye camera. In this paper, we assume that although each single eye only gets local information from its neighbors, the majority of the local motion classes can follow the global moving direction. With this assumption, the proposed model aggregates the voting of each single-eye-wise motion class to determine the class of the whole camera motion. Here, each individual local motion has identical weights, so the mode class of the local motion classifications becomes the final classification result. We call this process as the *voting step*, and the formulation of the *voting step* is as below,

(1) $${\mathrm{Class}}_{f}=argmax\sum_{i,j}M{\left(I_{k}\right)}_{i,j}$$

where $Class_{f}$ is the result of the final classification, $I_{k}$ is the *k*-th compound image pair, and $M{\left(I_{k}\right)}_{i,j}$ is a local classification result in one-hot vector form in dimension $n_{d}$.

### 3.4. Network Training

Until now, we have introduced the proposed ego-motion classification network for the compound eye camera. The proposed network is trained by reducing the *cross entropy loss* between the local classification distributions and the single-eye-wise ground truths. The *cross entropy loss* is defined as,

(2) $$Loss\left(I_{k}\right)=-\sum_{i,j}G_{s}\left(I_{k}^{i,j}\right)\log M{\left(I_{k}\right)}_{i,j},$$

where $I_{k}$ is the *k*-th compound image pair, $I_{k}^{i,j}$ is a single eye image in the *i*-th row and *j*-th column on $I_{k}$, $G_{s}$ is the single-eye-wise ground truth, and $M{\left(I_{k}\right)}_{i,j}$ is a local classification result of a single eye on the input $I_{k}$’s *i*-th row and *j*-th column. Note that only single-eye-wise motion classifications affect to the network training.

For training the proposed model, we use TensorFlow [28] which is a framework specialized in training deep neural network. The proposed network is optimized with Adam [29] with 0.0001 learning rate and 0.00001 weight decay coefficient for 50 epochs with the batch size of 256. These neural network training parameters are the only hyper-parameters for the proposed model. We basically followed the values of the ResNet training parameters [30] for ImageNet [31] dataset (initial learning rate = 0.1, weight decay coefficient = 0.0001, batch size = 256). We found out that the proposed model underfits with the learning rate of 0.1, so we empirically reduced the learning rate into 0.0001 to avoid the underfitting. We used a NVIDIA TITAN Xp GPU with 12GB memory for the training.

## 4. Experiments

We show the experimental details in this section. First, we introduce the compound image mapping method [10] which is used to transform the RGB images into compound images. Unfortunately, to the best of our knowledge, there is no publicly available dataset for compound eye ego-motion classification. Hence, we collected a new dataset, of which the details are described in Section 4.2. In the experiments, we have studied various configurations of the compound eye camera to understand the properties of the compound eye camera and to find an appropriate setting for ego-motion classification. Here, each configuration is determined by varying *S* and *r* values.

### 4.1. Compound Image Mapping

In this section, we introduce the compound image mapping method [10], of which the goal is to transform RGB images into the emulated compound images. We assume that the single eye images are uniformly deployed on a hemisphere surface, which is the typical compound eye configuration, and the RGB camera captured the far enough object so as we can consider the RGB image as a plane. We can consider a spherical coordinate system of which the origin is the same as the center of the hemisphere surface. Without loss of generality, we assume that the RGB image is captured with the camera that facing the direction of the z axis. The key of the compound image mapping procedure is to transform the image at view *a* (the RGB image) to a single eye image at view $b=\left(r,\theta_{b},\phi_{b}\right)$, where *r* is the radius of the hemisphere, θ is the polar angle, and ϕ is the azimuthal angle. To handle this, the homography $H_{ba}$ between the images at *a* and *b* is needed. $H_{ba}$ can be obtained as [32]

(3) $$H_{ba}=R_{ba}-\frac{t_{ba}n^{T}}{d},$$

where *n* is the normal vector of the image plane at view *a*, *d* is the distance between the camera and the image plane at view *a*, $t_{ba}$ is the translation vector from *a* to *b*, and $R_{ba}$ is the rotation matrix by which *b* is rotated in relation to *a*. Here, $R_{ba}$ can be obtained as follows:

(4) $$R_{ba}=\left[\begin{array}{ccc} 1 & 0 & 0\\ 0 & \cos(\theta_{b}) & -\sin(\theta_{b})\\ 0 & \sin(\theta_{b}) & \cos(\theta_{b}) \end{array}\right]\left[\begin{array}{ccc} \cos(\phi_{b}) & \sin(\phi_{b}) & 0\\ -\sin(\phi_{b}) & \cos(\phi_{b}) & 0\\ 0 & 0 & 1 \end{array}\right],$$

and $t_{ba}$ is calculated as

(5) $$t_{ba}=\left(r\sin\left(\theta_{b}\right)\cos\left(\phi_{b}\right),r\sin\left(\theta_{b}\right)\sin\left(\phi_{b}\right),r\cos\left(\theta_{b}\right)\right).$$

**Figure 5:**
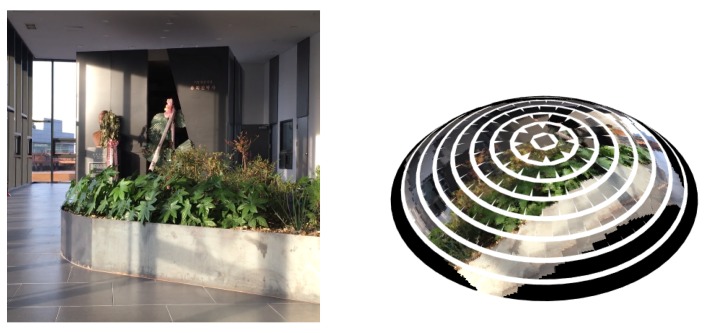
An example of RGB image and corresponding compound image constructed by the compound image mapping method from [10].

Finally, a pixel $p_{a}$ on the RGB image at view *a* is transformed to a pixel $p_{b}$ on the image at view *b* with

(6) $$p_{b}=K_{b}H_{ba}K_{a}^{-1}p_{a},$$

where $K_{a}$ and $K_{b}$ are intrinsic camera parameter matrices, and both $p_{a}$ and $p_{b}$ are in the homogeneous coordinates. For the single eye images, we assume that the intrinsic parameters are the same as the ones of the RGB camera. Note here that only valid pixels within the size of the single image are selected. An example of the proposed compound mapping is shown in Figure 5. In this way, we can simulate various configurations of compound eye structures.

### 4.2. Proposed Dataset

The newly proposed dataset was collected at inside and outside of a building in an university campus that includes scenes such as classroom, aisle, stairs, and terrace. We collected 80 videos which are captured by a moving RGB camera with a resolution of 1920×1080 pixels. In each video, the camera moves in one direction among the eight directions described in Section 3.2. The average number of frames in the 80 videos is 223. We applied the compound image mapping method [10] to these frames to emulate compound images. In this step, we can determine the single eye image size (*S*) of the emulated compound images. We have emulated compound images with various *S* (5, 10, 15, 20, 25, and 30) from a single RGB image source for analyzing the effect of various configurations. Analysis of the various configuration of the compound images will be described in Section 5.1.

**Figure 6:**
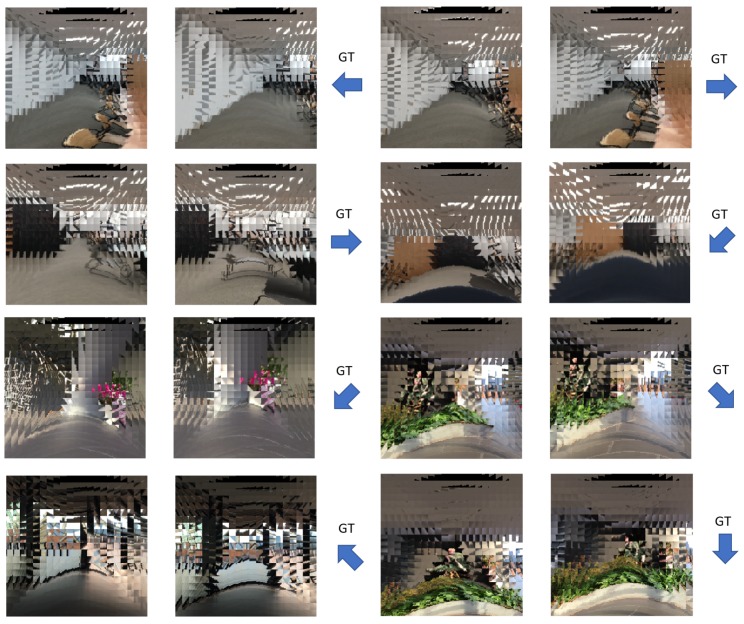
Some examples of the proposed dataset. Each sample consists of two consecutive compound images and their corresponding ground truth camera motion. The proposed dataset contains scenes of inside and outside of a certain building such as classroom, aisle, stairs, and terrace.

Each sample of the dataset consists of two compound images as source and target, and the corresponding ground truth camera motion class. The time interval between the source image and target image is 20 frames. In this manner, we have generated a total 13,348 number of training samples, and 2929 number of test samples for each single eye size from S=5 to S=30. Figure 6 shows some examples of compound image pairs and their corresponding ground truth motions in the proposed dataset.

**Figure 7:**
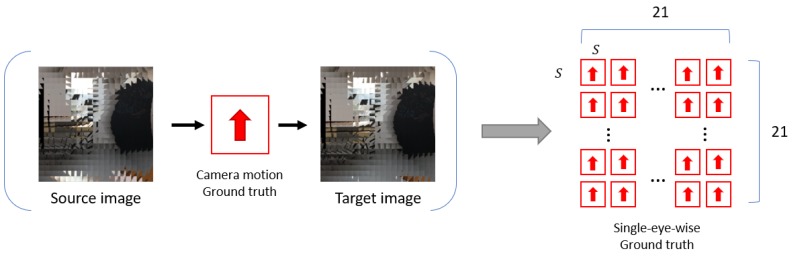
Single-eye-wise ground truth generation method for training the proposed ego-motion classification network. We assign the same motion as the ground truth camera motion in the proposed dataset to each of the $n_{l}\times n_{l}$ single eyes. With the assumption that the majority of single eyes capture the same motion as the ego-motion of the compound eye camera, the single-eye-wise ground truth data generated in this manner can be useful which considers the individual spatial information of single eyes, such as the proposed model.

In the proposed dataset, there are eight discrete directions of ground truth ego-motion; up, down, left, right, up-left, up-right, down-left, and down-right (see Figure 4). These ground truths are based on the movement of the entire compound eye camera. In the experiments, we expand these ground truth ego-motions to the ground truth of individual single eye motions (see Figure 7). The proposed ego-motion classification network is trained based on these single-eye-wise ground truth.

### 4.3. Baseline Algorithms

To verify that the proposed voting-based classification network is effective structure for compound images, we have compared the proposed scheme to two baseline algorithms. The first baseline is a classical block-based matching method [12] for ego-motion estimation, and the other one is a CNN-based algorithm which does not use the proposed voting-based classification.

**Figure 8:**
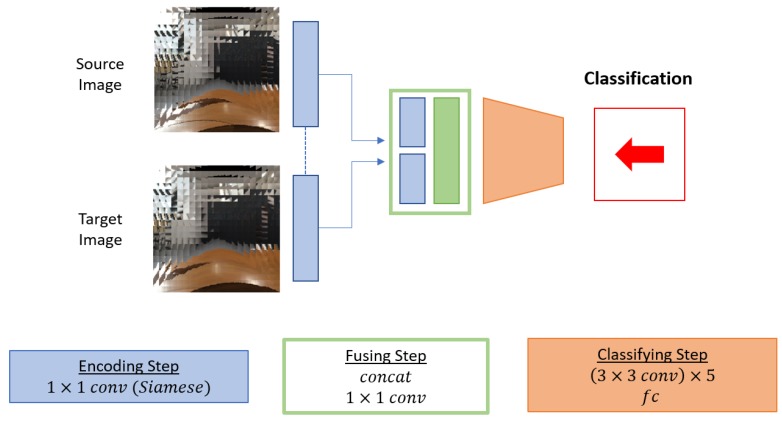
An overview of the baseline network. The baseline structure consists of three steps: the *encoding step*, the *fusing step*, and the *classifying step*. The *encoding step* and the *fusing step* are same as the proposed motion estimation network described in Figure 2. The *classifying step* consists of five 3×3 convolutional networks and two fully connected layers where three of the convolutional layers have stride of 2. The *classifying step* outputs one motion classification for the whole compound image rather than providing local classes for each single eye locations like the proposed network.

1. *Block-based matching algorithm.* In this algorithm, the source and target images are segmented into non-overlapping square blocks. With this setting, the algorithm finds the most matched target block for each source block. Here, mean square error (MSE) between two blocks is the criteria for the matching. We use exhaustive search to find the matching block, since it guarantees the optimal solution. The displacement of the matching pairs becomes the motion vector of the source block, and we discretize it into eight directions for the ego-motion classification described in Figure 4.
2. *CNN without voting.* The other baseline algorithm is a CNN-based algorithm which is similar to the proposed algorithm but compute the ego-motion of the whole compound eye camera directly instead of classifying individual single eye motions. The baseline network structure is same with the proposed network up to the *encoding step* and the *fusing step*, but after that, it has the *classifying step* instead of the *gathering step* and the *voting step* (see Figure 8). The *encoding step* consists of 3×3 convolutional layers, some of which have stride of 2, and fully connected layers. Therefore, the *encoding step* integrates the features of all single eyes into a vector form rather than maintaining individual features and spatial relationships of the single eye images. The output of the *encoding step* is a distribution of the discretized motion classes over the entire FOV of the compound image. Note that all convolutional layers in the *gathering step* have stride of 1, which keeps the shape of feature map in each layers same. The detailed architecture of the baseline network is summarized in Table 2.

### 4.4. Evaluation Metrics

For the evaluation metrics, we use the accuracy of classification and the *confidence* value. The classification accuracy of the model is measured by comparing the results of the final classification, i.e., the results of the *voting step* in case of the proposed model, and the ground truth of the whole image motion. The *confidence* value is a value that represents the average percentage of the vote of the most frequently chosen direction in the local motion classification of a sample. The definition of the *confidence* is as below,

(7) $$\mathrm{confidence}\left(I_{k}\right)=\frac{\max\sum_{i,j}M{\left(I_{k}\right)}_{i,j}}{n_{l}^{2}}$$

where $I_{k}$ is the *k*-th compound image pair, $M{\left(I_{k}\right)}_{i,j}$ is a local classification result in one-hot vector form in dimension $n_{d}$, and $n_{l}$ is the size of the vectorized compound image. The *confidence* represents how many single-eye-wise local classifications agree the final classification. It also can be understood as the accuracy of the local classifications itself.

## 5. Results

In this section, we report the performance of the proposed scheme on the proposed dataset with various configurations of *S* and *r*. These two values are the main factors of determining the trade-off between the accuracy and the computational cost. The configurations we have explored are the combinations of *S* from 5 to 30, and *r* from 1 to 5. Note that the *S* value is determined in the compound image mapping step and fixed after that, so *S* and *r* values can be changed independently. Table 3 and Table 4 represent the classification accuracy and *confidence* of the proposed model with various configurations. Note that all components in Table 3 and Table 4 are the performances of the proposed model with different settings of parameter *S* and *r*. From the results of Table 3 and Table 4, we can find that the accuracies of the final classifications are higher than the *confidence* values in the same configuration. These gaps demonstrate the validity of our strategy for designing local classification network, which focuses on the efficiency rather than the accuracy, with the expectation that the accuracy of the classification would be improved by the *voting step*.

### 5.1. Analysis of the Various Configurations

In this section, we analyze the effect of the *S* and *r* value to the model performance. The size of a single eye image, *S*, is related to the details of a specific direction view. A bigger single eye image contains more details, but it also requires more memory and computational cost for the *encoding step*. The receptive field size of the local classification, *r*, also has similar trade-off between more features and the computational cost. The local classification network with bigger receptive field gathers more visual features from wider area, but also its memory requirement and computational cost are increased due to the additional convolutional layers.

**Figure 9:**
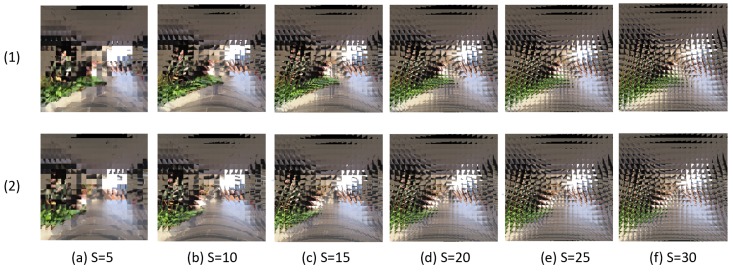
Examples of pairs of compound images with various *S*. (1) and (2) represent two sequential scenes in the dataset. (**a**–**f**) show the compound images of different *S* from 5 to 30 at the same scene.

**Figure 10:**
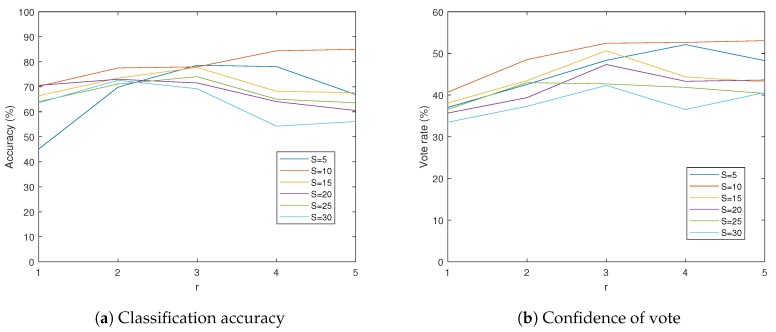
Results of the proposed compound eye ego-motion classification network in varying *S* and *r*. S=10 achieves the highest accuracy and *confidence* value when *r* is fixed. Similarly, r=3 achieves the highest accuracy and *confidence* value when *S* is fixed, except for the case S=10 which has best performance at r=5.

From the results of Table 3 and Table 4, we find a tendency that increasing accuracy by increasing *S* and *r* affect reversely at some points. That is, to achieve a reasonable accuracy, *S* and *r* should be neither too small nor too big. It is clear that the small *S* lacks the information of each single eye image, and the small *r* lacks information of the neighboring single eye images. The reason of the low accuracy with big *S* can be understood by comparing visualized compound images with different *S*. As seen in Figure 9, the bigger *S* makes the single eye images more overlap to each other. Since a single eye image is encoded into a vector form, the spatial features inside the single eye images are lost in the *encoding step*. Therefore, overly overlapped neighboring single eye images are encoded into similar vectors, which make it hard to distinguish them. This similarity due to the big *S* eventually disturbs the ego-motion classification since the local camera motion is classified by comparing differences of neighboring features. Through the experiments, we find that the model with S=10 achieves the best performance (see Figure 10).

**Figure 11:**
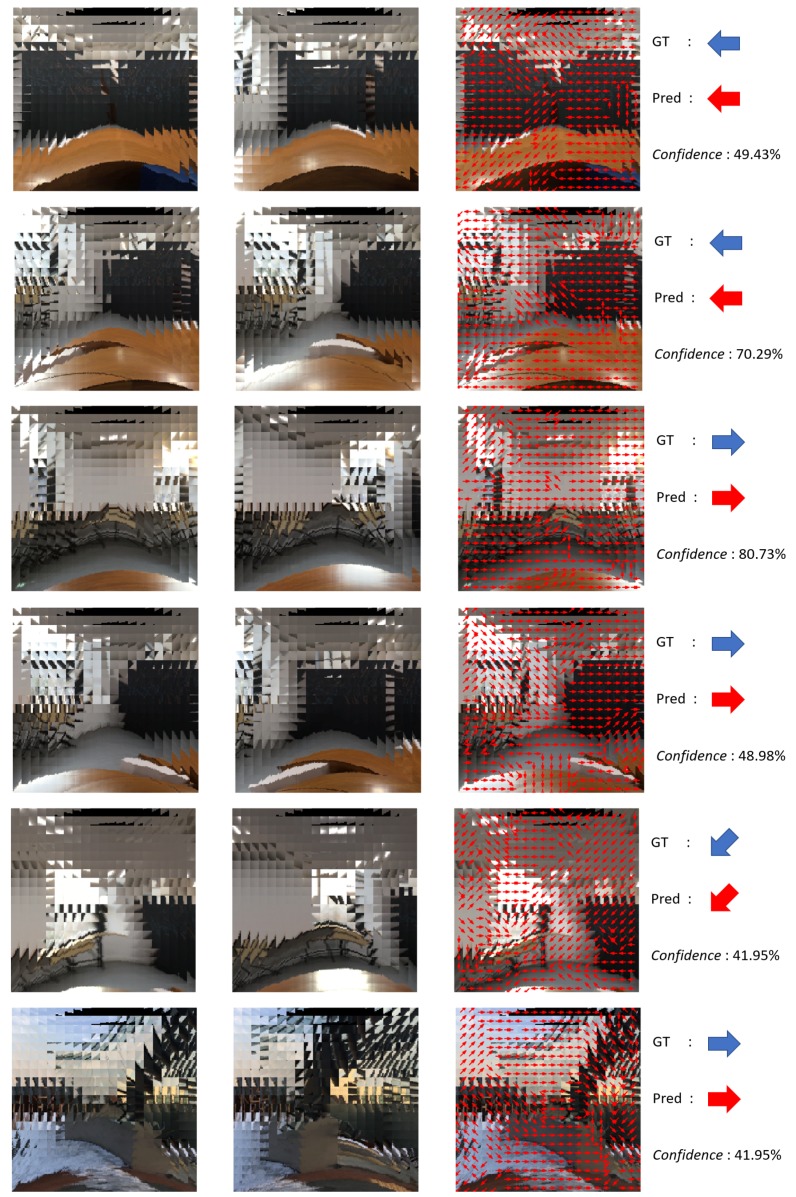
Some successful results of the proposed ego-motion classification network.The first, and the second columns are two consecutive compound images. The third column visualizes local classifications on each single eye position. The last column shows the ground truth and classified result of camera motion and *confidence* value.

In case of the big *r*, the local classification is obtained based on the features inside the wide FOV. This can be an issue when there exist multiple features which are clues of the different camera motions in the same local FOV, since these features cancel each other’s influence to the classification. In other words, the distribution of the single-eye-wise classification becomes ambiguous, so it cannot represent clear tendency of the camera motion of the given single eye image position. Therefore, the *r* value should also be selected carefully, not too small or big. Through the experiments, we find that r=3 is the most proper point, except for the case of S=10 where the configuration of r=5 achieves the highest accuracy and the *confidence* value (see Figure 10). As a result, in our experiments, the model with S=10,r=5 shows the highest accuracy of 85.0% and the *confidence* value of 53.1%. Figure 11 shows some successful examples of the proposed algorithm.

### 5.2. Computational Cost and Memory Storage

In this section, we report the computational cost and memory storage requirement of the proposed algorithm. Table 5 shows the number of training parameters of the proposed model in various configurations. Table 6 shows the required floating point operations per second (FLOPs) to infer a sample with $n_{l}=21$. Since the network with bigger *r* has more convolutional layers, *r* has more influence than *S* in determining the complexity of the model including both number of parameters and FLOPs. In the case of the model with S=10,r=5, the number of parameters in the network is about 0.9M and 1.8M FLOPs are required for an inference.

Table 7 ilustrates the number of parameters and FLOPs of the popularly using CNN-based image recognition networks. Our proposed model with S=10,r=5 is much lighter compared to the other conventional CNNs, where MobileNetV2 [33] and ShuffleNetV2 [34], which are designed to be light-weight are included. Note that the networks in Table 7 takes 224×224 size RGB image as an input ( $R^{224\times 224\times 3}$), which is the similar scale to the compound image with $n_{l}=21$, and S=10 ($R^{3S^{2}\times n_{l}^{2}}$). Although the data types and tasks of these networks are not identical to the proposed network, this comparison gives us an intuition that our model can be claimed as light-weight among the CNN-based models.

### 5.3. Comparison with Baseline Algorithms

In this section, we compare the proposed algorithm with the two baseline algorithms described in Section 4.3. We make comparisons of these algorithms on two cases: compound image version and 2D RGB image version of the proposed dataset. Table 8 illustrates the results of each algorithm on the two cases. Note that the block-based algorithm can be easily extended to the compound image domain by using single eye images as blocks. We use this modified version of the block-based algorithm in the experiments on the compound images. We also note that the CNN-based baseline network is designed to have 0.9M number of parameters for the fair comparison to the best configuration of our proposed model (S=10,r=5). The performances of these baseline algorithms are evaluated by the same measures as the proposed algorithm; discretized ego-motion classification accuracy and the *confidence* score.

**Figure 12:**
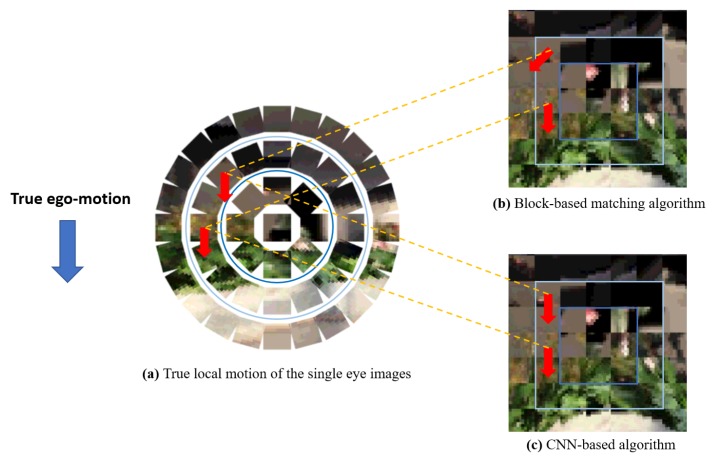
An example of the geometric distortion when mapping a compound image to 2D plain surface. (**a**) True local motion of the single eye images on the compound image hemisphere surface. (**b**) Local classification via the block-based matching algorithm. Since the upper single eye image is rotated when projected to the 2D plane, the direction of its matching block is also moved. (**c**) Local classification via the CNN-based algorithm. The CNN learns how to classify true motion of each single eye from the distorted compound image on the 2D plane.

In case of the block-based matching algorithm, the ego-motion classification accuracy in 2D RGB image domain is 46.5% and the *confidence* is 28.6%, which are inferior to the proposed algorithm (see Table 8). In compound image domain, compound images are projected to the 2D plane to construct structured square image form to apply the block-based algorithm (Figure 12b). On the 2D projected compound image, each single eye image role as a small block of the block-based algorithm. With these data, the algorithm shows low accuracy of 19.3% and *confidence* of 22.3%, which is only slightly better than the expected performance of the random choice. This collapse of the performance is due to the geometric distortion of single eye locations when mapping hemisphere compound eye surface on 2D plane. Figure 12 shows an example of how the mapping geometric distortion disturbs the block-based matching algorithm, which is designed for 2D image domain. For example, two single eye images with the same ego-motion on the compound eye hemisphere surface (Figure 12a) can be turned in different directions on the 2D plane (Figure 12b). Since the block-based matching algorithm uses pixel values of blocks for the matching, it does not able to consider this geometric distortion. Therefore, many motion vectors of single eye images obtained by block-based matching algorithm are deviated from the true ego-motion even if it finds the most matching pairs, which leads to the poor classification accuracy. On the other hand, CNN-based algorithms can avoid this mis-alignment problem since they exploit trained feature space encoding for classifying single eye image motion (Figure 12c). That is, through the training process, the neural networks learn how to encode data to the feature space which can represent the true ego-motion of a single eye image by gathering the neighboring features.

In the case of the CNN-based baseline algorithm without voting, the classification accuracy in 2D RGB image domain and compound image domain are 46.7% and 64.1%, respectively, which are lower than the proposed algorithm. Note that the *confidence* score is not applicable to this baseline network since the CNN without voting does not output individual motion class of each single eye images. In addition, we find that the CNN without voting is more likely to overfit to the training dataset than the proposed network, so we adopts the early stopping technique to avoid the overfitting problem.

## 6. Conclusions

In this paper, we have proposed a CNN-based light-weight compound eye ego-motion classification framework for the first time, to the best of our knowledge. To fully utilize the unique structure of the compound eye that perceive scenes by gathering small images from a number of single eyes, the proposed algorithm makes the final classification by aggregating the local classifications from each single eye image. We have explored various configurations by changing single eye size (*S*) and receptive field size of local classification (*r*) to find an appropriate compound eye camera configuration for the future hardware development. We have demonstrated that the proposed model shows superior performance than the baseline algorithms, which verifies the effectiveness of the proposed model. The proposed network have much fewer number of training parameters and FLOPs compared to the popularly used CNN architectures for image recognition in RGB image domain. A limitation of the proposed scheme is that it is designed for classifying only 2D directions. Knowing the forward and backward movement is also important for robot localization and 3D environment recognition. We leave the research on the 3D ego-motion classification which can aware the forward-backward ego-motion as well as 2D motions, for the compound images as future work.

## Figures and Tables

**Table 1:** Detailed structure of the proposed ego-motion classification network. Each layer in the row has output dimension of *c*, stride of *s*, zero padding of *p*, and is repeated *n* times. Since all the convolutional layers in the proposed network have stride of 1, and all the 3×3 convolutional layers have zero padding of 1, the size of the compound eye image ($n_{l}^{2}$) is preserved until the output. We also note that since all the layers with training parameters are convolutional layers, the proposed ego-motion classification network can be applied to any size of $n_{l}$.

Input	Operator	*c*	*s*	*n*	*p*
$n_{l}^{2}\times 3$ S${}^{2}$	conv2d 1 × 1	16	1	1	0
$(n_{l}^{2}\times 16)\times 2$	concat	32	-	1	-
$n_{l}^{2}\times 32$	conv2d 1 × 1	128	1	1	0
$n_{l}^{2}\times 128$	conv2d 3 × 3	128	1	r	1
$n_{l}^{2}\times 128$	conv2d 1 × 1	8	1	1	0

**Table 2:** Detailed structure of the CNN based baseline network without voting. Each layer in the row has output dimension of *c*, zero padding of *p*, and stride of *s*. In contrast to the proposed network described in Table 1, the baseline network contains some convolutional layers with stride 2, which reduce the output feature map size.

Input	Operator	*c*	*s*	*p*
$21^{2}\times 3$ S${}^{2}$	conv2d 1 × 1	16	1	0
$(21^{2}\times 16)\times 2$	concat	32	1	-
$21^{2}\times 32$	conv2d 1 × 1	64	1	0
$21^{2}\times 64$	conv2d 3 × 3	64	2	1
$11^{2}\times 64$	conv2d 3 × 3	64	2	1
$6^{2}\times 64$	conv2d 3 × 3	64	1	1
$6^{2}\times 64$	conv2d 3 × 3	128	2	1
$3^{2}\times 128$	conv2d 1 × 1	128	1	0
$3^{2}\times 128$	fc	512	-	-
512	fc	8	-	-

**Table 3:** Compound eye ego-motion classification accuracy (%).

	S	5	10	15	20	25	30
r							
1		45.1	70.2	66.4	70.6	64.1	63.6
2		69.8	77.5	73.5	73.0	71.1	72.6
3		78.6	78.0	77.7	71.5	74.0	69.2
4		78.1	84.4	68.2	64.1	65.0	54.3
5		66.9	**85.0**	67.7	60.5	63.6	56.1

**Table 4:** Average percentage of the vote of the most frequently chosen direction in the *voting step* (=*confidence*, %).

	S	5	10	15	20	25	30
r							
1		37.0	40.7	38.1	35.7	36.6	33.5
2		42.6	48.5	43.5	39.4	43.0	37.3
3		48.3	52.5	50.6	47.3	42.7	42.3
4		52.1	52.7	44.4	43.3	41.9	36.5
5		48.3	**53.1**	43.2	43.6	40.4	40.6

**Table 5:** Number of parameters of various configurations of the proposed model.

	S	5	10	15	20	25	30
r							
1		303 K	306 K	312 K	321 K	332 K	345 K
2		450 K	454 K	460 K	468 K	479 K	492 K
3		598 K	601 K	607 K	616 K	627 K	640 K
4		745 K	749 K	755 K	763 K	774 K	787 K
5		893 K	897 K	903 K	911 K	922 K	935 K

**Table 6:** FLOPs of various configurations of the proposed model.

	S	5	10	15	20	25	30
r							
1		0.605 M	0.612 M	0.623 M	0.640 M	0.662 M	0.688 M
2		0.899 M	0.907 M	0.918 M	0.935 M	0.957 M	0.983 M
3		1.19 M	1.20 M	1.21 M	1.23 M	1.25 M	1.29 M
4		1.49 M	1.50 M	1.51 M	1.53 M	1.55 M	1.57 M
5		1.78 M	1.79 M	1.80 M	1.82 M	1.84 M	1.87 M

**Table 7:** Comparison with some popular CNN-based image recognition algorithms. Note that MobileNetV2 [33] and ShuffleNetV2 (×1) [34] are the networks which are designed to be light-weight.

	Number of Parameters	FLOPs
AlexNet [35]	60 M	720 M
VGG16 [36]	138 M	153 G
ResNet50 [30]	25 M	4 G
MobileNetV2 [33]	3.4 M	300 M
ShuffleNetV2 (×1) [34]	2.3 M	146 M
**Ours (S=10,R=5)**	**0.9 M**	**1.8 M**

**Table 8:** Comparison with baseline algorithms.

	2D RGB Image		Compound Image	
	Accuracy (%)	Confidence (%)	Accuracy (%)	Confidence (%)
Block-based matching	46.5	28.6	19.3	22.3
CNN w/o vote	46.7	-	64.1	-
CNN w/ vote (ours)	88.0	49.3	85.0	53.1
